# A Novel Fiber-Optical Fabry–Perot Microtip Sensor for 2-Propanol

**DOI:** 10.3390/s25072178

**Published:** 2025-03-29

**Authors:** João M. Leça, Paulo Antunes, Florinda M. Costa, António J. S. Teixeira, Marta S. Ferreira

**Affiliations:** 1 Institute for Nanostructures, Nanomodelling and Nanofabrication (i3N) and Physics Department, University of Aveiro, Campus Universitário de Santiago, 3810-193 Aveiro, Portugal; pantunes@ua.pt (P.A.); flor@ua.pt (F.M.C.); marta.ferreira@ua.pt (M.S.F.); 2 Institute of Electronics and Informatics Engineering of Aveiro (IEETA), Department of Electronics, Telecommunications and Informatics, University of Aveiro, Campus Universitário de Santiago, 3810-193 Aveiro, Portugal; ajst@ua.pt

**Keywords:** optical fiber sensor, Fabry–Perot interferometer, polymer microtip, 2-propanol, guided photopolymerization, biomarker

## Abstract

2-Propanol in the gaseous phase of clinical samples can serve as a biomarker for disease diagnosis. In this context, a novel fiber-optic Fabry–Perot (FP) interferometric sensor with a microtip structure was developed using the light-guided induced polymerization technique. The optical fiber sensor (OFS) with the best performance, measuring approximately 15 µm in length, exhibited good sensitivity to 2-propanol, with a response of −71.1 ± 2.1 pm/ppm. Additionally, it demonstrated good stability, with a maximum standard deviation of 0.15 nm and an estimated resolution of 3.18 ppm. The good sensitivity and ease of fabrication of this OFS highlight its potential for biomedical applications, particularly in non-invasive disease detection, given the role of 2-propanol as a biomarker for various health conditions.

## 1. Introduction

Volatile organic compounds (VOCs) are commonly found in everyday activities and in various industries such as chemical production, industrial safety, and food processing, among others. Particularly, in the biomedical field there is an increasing need for developing highly sensitive and precise detection methods to measure VOCs at low concentrations, as these characteristics are essential for using VOCs as biomarkers, enhancing their utility in disease diagnostics [1,2].

Biochemically, VOCs are metabolic byproducts of the human body. They can be detected in the gaseous phase of clinical samples, such as breath, saliva, sweat, urine, and feces. These low-molecular-weight compounds form a “metabolic signature” that can be altered by pathological conditions, making certain VOCs potential biomarkers for disease diagnosis [1,2,3]. Fluctuations in their levels may reflect changes in metabolic pathways, such as disruptions in protein and enzyme activity or gene regulation [1,4]. These variations can be detected using techniques like gas chromatography–mass spectrometry [5], electronic noses [6], and OFSs [7], among others.

Specific VOCs show potential as diagnostic markers for various diseases when their levels differ from typical ranges. Among these, 2-propanol (isopropanol), ethanol, and acetone are frequently highlighted in volatomics research focused on prevalent health conditions such as type II diabetes and cancer [1,4]. 2-Propanol, a secondary alcohol, has been identified as a promising biomarker with different associations with several diseases across different biological samples. Indeed, fluctuations in levels of 2-propanol in breath may be associated with various cancers [8]. Elevated levels of 2-propanol in urine have been linked to type II diabetes [9], whereas lower concentrations are found in cases of malignant biliary strictures [3]. On the other hand, in fecal samples, increased levels of 2-propanol can be correlated with colorectal cancer [10]. These observations highlight the potential of 2-propanol as a biomarker for disease detection, either used individually or in combination with other VOCs, as part of an integrated biomedical system designed for specific diagnostic objectives.

OFSs are widely recognized for their numerous advantages, including their light weight, robustness, immunity to electromagnetic interference, electric passiveness (no electric signal at the measuring point), and biocompatibility. Additionally, OFSs are compact and can be functionalized to enhance their sensitivity and/or selectivity to specific targets, such as 2-propanol, by utilizing materials with a high affinity for this compound. The cost of interrogation systems for OFSs remains the main challenge in their widespread adoption. This issue has been targeted by the scientific community, which is actively developing solutions to make these systems more affordable [11,12].

Detecting VOCs using OFSs has gained significant attention due to their potential for real-time and accurate measurements. Various manufacturing approaches and sensing materials, such as polydimethylsiloxane, polymethyl methacrylate, and zeolite films, have been reported in the literature [13,14,15]. However, the development of viable OFSs for VOCs detection remains challenging. Key issues include achieving high sensitivity and selectivity while mitigating signal instability. Although OFSs can offer high sensitivity, they can also be limited by the complex dynamic nature of VOC mixtures and cross-sensitivity to other compounds. Further advancements are required to develop more reliable, sensitive, and practical OFS-based VOC sensors that can effectively address these challenges and meet the demands of real-world applications. Among the interferometric sensors, FP designs are particularly targeted to address these challenges due to their high sensitivity and potential for advanced customization [7,16,17].

One of the several approaches to developing microtips at the extremities of optical fibers involves manufacturing them through light-guided photopolymerization. This technique involves the injection of UV light into one end of the optical fiber. The light is guided along it, and when the light exits the fiber at the other end, which is in contact with a photopolymerizable polymer, a tip is formed. By adjusting various parameters, such as the intensity and irradiation time, fiber type, and photopolymerizable material, among others, it is possible to control the structure that is created [18,19,20]. This method does not require advanced techniques like two-photon polymerization, which need more complex and expensive instrumentation [21]. The structures generated by the light-guided photopolymerization technique gain the three-dimensional shape of the light propagation, which strongly depends on the optical fiber used. The resulting structures are considerably different from those obtained when the adhesive curing is performed with light not guided by the fiber but through external radiation [22]. This type of structure has been used as sensing elements for various parameters, including the refractive index [23,24], humidity [25], temperature [26], and others. Additionally, a polymer microtip on a multimode fiber (MMF), combined with an optical backscatter reflectometer, has been used to study VOCs as a condensed material threshold sensor [27].

Given the potential of 2-propanol as a biomarker for diseases and the demand for advancements in VOC sensor technology, this research aims to create a cost-efficient, simple-to-manufacture, and reproducible sensor for highly sensitive detection of 2-propanol. The sensor will utilize an FP interferometer at the end of an MMF, fabricated through light-guided photopolymerization. To the best of our knowledge, this is the first example of light-guided induced polymerization being used to create an FP interferometer for the detection of 2-propanol. This research aims to contribute to the advancement of novel detection devices for biomedical applications or other fields where VOC detection is of significant interest.

## 2. Sensing Principle and Fabry–Perot Microtip Manufacturing

To meet the critical requirements for OFS-based 2-propanol detection, an ultra-short FP interferometer located at the tip of an MMF is proposed. This section presents its principles and the fabrication process.

### 2.1. Sensing Principle

**Figure 1:**
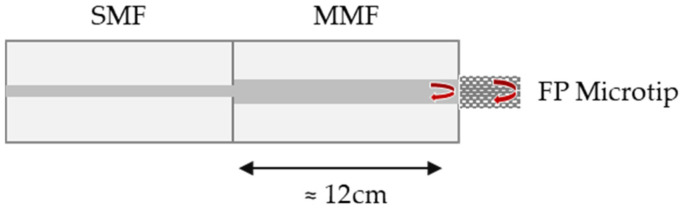
Schematic diagram of the fiber-optic Fabry–Perot (FP) microtip and the two reflective surfaces. SMF—single-mode fiber, MMF—multimode fiber.

The schematic diagram of the FP interferometric sensor is depicted in Figure 1. The FP cavity is formed between two reflective surfaces: the end face of an MMF and the end face of the microtip.

The reflection optical spectrum of the sensor can be described by the following equation:

(1) $$I\left(\lambda \right)=I_{1}\left(\lambda \right)+I_{2}\left(\lambda \right)+2\sqrt{I_{1}\left(\lambda \right)I_{2}\left(\lambda \right)}\cos\left(\frac{4\pi n_{eff}L}{\lambda }\right)$$

In this context, *I*_1_(*λ*) and *I*_2_(*λ*) refer to the reflected optical intensities from the fiber end and the microtip, *λ* is the light wavelength, $n_{eff}$ is the effective refractive index of the FP interferometer, and *L* is the cavity length. As VOC molecules are absorbed into the microtip, the diffusion within the 3D polymer structure leads to swelling effects and refractive index changes, which are anticipated to result in a shift in the resonant dip [28].

### 2.2. Sensor Fabrication Process

The light-induced microtip fabrication method uses a distinct approach. It starts by introducing ultraviolet (UV) radiation into one end of an optical fiber. As the light travels through the fiber and exits from the opposite end, it initiates the polymerization reaction in the photosensitive polymer solution. This interaction results in the formation of a microscopic tip structure at the end of the fiber.

**Figure 2:**
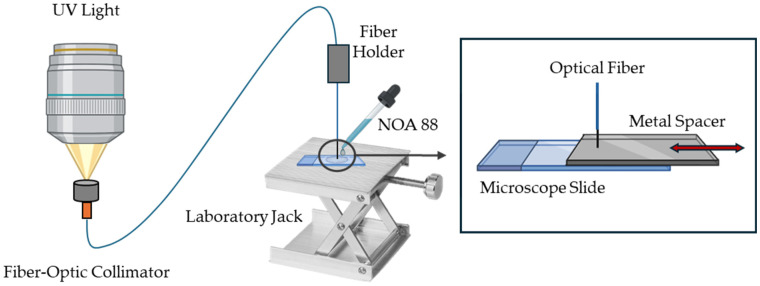
Schematic fabrication process diagram of the light-guided induced microtip. NOA 88—Norland Optical Adhesive 88.

Figure 2 presents the schematic diagram of the fabrication process. Briefly, the mounted UV LED (Thorlabs M365LP1, Newton, NJ, USA) was connected to an adjustable collimation adapter (Thorlabs SMF321, Newton, NJ, USA) and mounted vertically, approximately 10 cm above a fiber-optic collimator with a pigtail adapter. The light was focused, ensuring maximum light transmission, into the fiber-optic collimator and subsequently guided through a silica step index MMF optical fiber FP5FC pigtail Easystrip, 50/125 µm (all4fiber, Vienna, Austria). The tip of the optical fiber was stripped and cleaved using a high-precision optical fiber cleaver, then fixed and aligned in the fiber holder.

With the help of a laboratory jack, the fiber tip was positioned close and perpendicular to a microscope slide. The distance between the fiber tip and the slide determined the length of the microtip. To maintain relative control over this distance, a metal spacer was used with a thickness equal to the desired length of the microtip. In this study, three sensors with different microtip lengths were manufactured using different metal spacers. A droplet of photopolymerizable polymer Norland Optical Adhesive 88 (NOA 88) from Norland Products (Jamesburg, NJ, USA) was then placed between the fiber tip and the slide, and the UV light was turned on at maximum irradiance, 17.6 µW/mm² (according to the manufacturer), for 10 s to proceed with the photopolymerization process. The excess adhesive was washed off with distilled water, and the polymer was cured at 50 °C for 12 h, as recommended by the manufacturer [29].

The NOA88 is a liquid adhesive with mercapto-esters and triallyl isocyanurate in its composition, commonly used in optical applications, that cures upon exposure to ultraviolet light. It is optically compatible, with strong adhesive properties, and is able to generate precise and durable structures. Despite its favorable characteristics, only one polymer with some similarities, NOA 81, from the same supplier, has been used as a sensing material in an OFS for VOCs detection [30].

**Figure 3:**
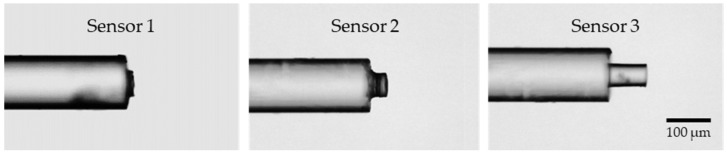
Microscope images of the 3 sensors developed at 100× magnification. The cavity length of sensor 1 ≈ 15 µm, of sensor 2 ≈ 40 µm, and of sensor 3 ≈ 89 µm.

After curing, the tips of the three manufactured sensors were examined under a microscope with 100× magnification (Figure 3). The cavity length for sensor 1 is approximately 15 µm, for sensor 2, 40 µm, and for sensor 3, 89 µm, achieved using metal spacers with a thickness of 20, 50, and 100 µm, respectively. Although the microtip length can be predicted with some degree of control, the fabrication process could be further improved to enhance the precision of the obtained cavity lengths. Finally, a section of approximately 12 cm containing the sensor microtip was sliced from the MMF optical fiber and spliced into a single-mode fiber (SMF) pigtail, enabling the connection to an optical acquisition system. After implementing initial adjustments to the sensor fabrication setup, it was verified that the process was straightforward and userfriendly.

## 3. Sensor Measurements and Characterization

**Figure 4:**
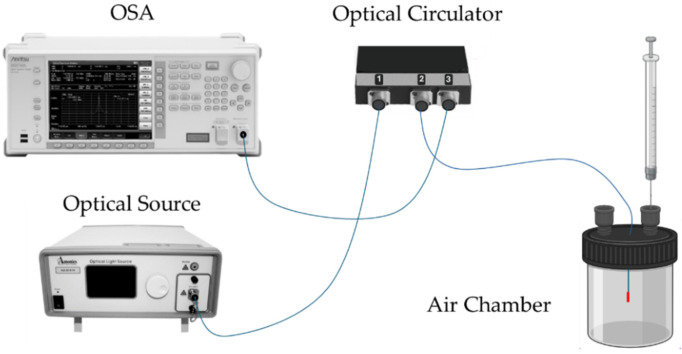
Schematic diagram of the sensor interrogation setup. OSA—optical spectrum analyzer.

The developed sensors were interrogated with a standard optical reflection setup, as depicted in Figure 4. In this configuration, the optical source was connected to port 1 of an optical circulator, the OFS to port 2, and the optical spectrum analyzer (OSA, Anritsu MS9740A, Kanagawa, Japan) to port 3. A broadband optical source (Amonics LS-CL-17-B-FA, Hong Kong) with a center wavelength of 1570 nm and an 80 nm bandwidth was used for the experiments. The sensor response was recorded using the OSA, operating with a resolution of 0.5 nm.

All experiments were conducted at room temperature, approximately 24 °C, with an environmental relative humidity variation from 50% to 65%. Initially, both access points of the air chamber were opened, and a small airflow was injected into one of the access points. This procedure was repeated between each sample analysis. The desired VOC gas concentrations were achieved by evaporating precise volumes of the respective compounds inside the isolated air chamber with a fixed volume of 100 cm^3^. It is also important to mention that, after injecting the VOC, the system was stabilized for 10 min before we recorded the corresponding spectrum. The cleaning step with atmospheric air lasted 20 min before we proceeded to the next sample. All the developed sensors were assessed for 2-propanol, ethanol, and acetone, with their concentrations varying between 0 and 100 ppm in increments of 25 ppm.

## 4. Results and Discussion

### 4.1. Overall Characterization of the Fabricated Sensors

**Figure 5:**
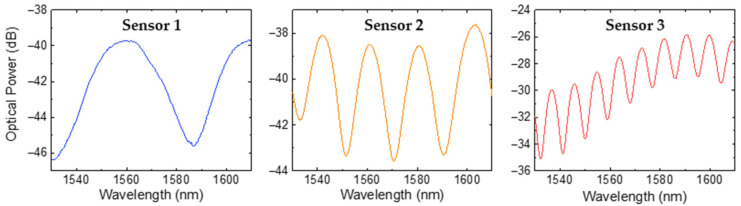
Reflection spectra of the FP microtip sensors formed by NOA88 adhesive when exposed to air.

The results of the three OFSs in response to air are presented in Figure 5. It can be verified that the OFSs exhibited an interferometric pattern characteristic of a two-wave FP interferometer. For simplicity, a two-wave interferometer model was applied, allowing us to calculate the effective refractive index, *n*_eff_, of the photopolymerized microtip using the following equation:

(2) $$n_{\mathrm{eff}}=\frac{\lambda_{1}\lambda_{2}}{2L_{FP}\Delta \lambda },$$

where *λ*_1_ and *λ*_2_ are the wavelengths of two adjacent peaks, with ∆*λ* = *λ*_2_ − *λ*_1_ representing the free spectral range and *L*_FP_ denoting the cavity length of the FP interferometer. For the three sensors, the average refractive index obtained at 1550 nm was 1.55 ± 0.04, which is close to the value provided by the manufacturer (1.56, at 589 nm) [29].

The reflected signal visibility (*V*) was calculated according to Equation (3):

(3) $$V=\frac{I_{max}-I_{min}}{I_{max}+I_{min}}$$

where *I*_max_ and *I*_min_ are the maximum and minimum intensity of the interference pattern, respectively. The calculated visibility values for the optical spectra were 63% for sensor 1, 51% for sensor 2, and 39% for sensor 3. The observed decrease in reflected signal visibility with increasing FP cavity length can be attributed to higher cavity losses, including beam divergence and material absorption [31,32].

### 4.2. Performance of Sensors in 2-Propanol Detection

**Figure 6:**
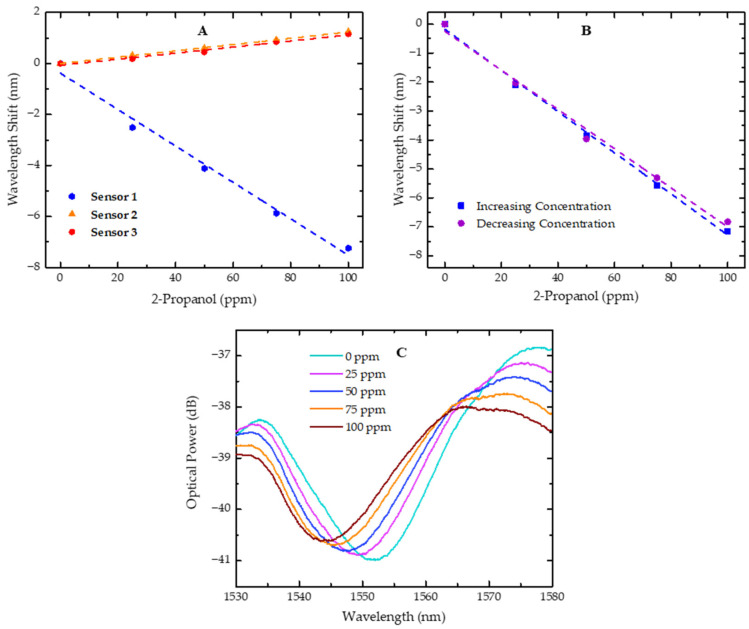
Wavelength shift of sensors in response to variations in 2-propanol concentration. (**A**) Wavelength shift for sensors 1, 2, and 3 with 2-propanol concentrations increasing from 0 to 100 ppm; (**B**) wavelength shift of sensor 1 for 2-propanol at increasing (0 to 100 ppm) and decreasing (100 to 0 ppm) concentrations; (**C**) plot of the resonant dip evolution for sensor 1 with increasing 2-propanol concentrations, ranging from 0 to 100 ppm. Dashed lines represent the linear fit applied to the experimental data.

All the sensors were characterized for 2-propanol under the same experimental conditions, in a concentration range between 0 and 100 ppm, a range typically found in urine samples [3], with increments of 25 ppm, and the results are shown in Figure 6A.

With the obtained results, it is possible to verify that, in sensor 1, there was a shift in the resonant dip towards shorter wavelengths (blue shift) when the concentration of 2-propanol was increased in the gas phase, with a sensitivity of −71.1 ± 2.1 pm/ppm. On the contrary, when sensors 2 and 3 were exposed to increasing concentrations of 2-propanol, the analyzed dip shifted towards longer wavelengths (red shift) with sensitivities of 12.4 ± 0.2 pm/ppm and 11.9 ± 0.1 pm/ppm, respectively. This result can be explained by the diffusion dynamics of small molecules into polymers.

By real-time tracking of resonant wavelength shifts in glassy polymer microspheres, Wang et al. [28] observed that the polymer behavior depends on the VOC diffusion stage, which is influenced by the polymer volume. These shifts in resonant wavelength, occurring in opposite directions, were noted as the material transitioned through different interaction phases, such as surface adsorption, outer-layer-limited diffusion, and saturation. In this work, the different sensors exhibited similar shifts in opposite directions, depending on the FP length, which may be due to the volume of the polymer that directly governed the interaction phase in which the spectra were collected, considering that the experimental data were always collected after 10 min of stabilization. Thus, it can be inferred that these different interaction phases depend on the FP length, distinctly affecting the swelling effects and refractive index of the polymer, ultimately influencing the shift tendency of the resonant dip.

The resonant dip in the spectrum arises from destructive interference, where the reflected waves from the two surfaces of the FP sensor interfere out of phase, leading to an optical power loss at specific wavelengths. This behavior can be observed for sensor 1 in Figure 6C, along with its evolution towards shorter wavelengths as the concentration of 2-propanol increases.

Given that our sensor microtip is composed of a polymer, it is important to study hysteresis effects, as they can have a significant impact on the measurement [33]. To assess this effect, the wavelength shift of one dip was evaluated for sensor 1. After the sensitivity test with increasing concentrations of 2-propanol from 0 to 100 ppm, the same test was repeated, but with decreasing concentrations, from 100 to 0 ppm, following the same procedure.

The results are presented in Figure 6B and the sensitivities estimated from the applied linear fits are similar and fall within the sensor’s sensitivity errors. This observation indicates that the hysteresis effect does not significantly affect the sensor performance in detecting 2-propanol over the range of concentrations studied. These results also suggest that the slight airflow and waiting time between measurements are adequate.

### 4.3. Cross-Sensitivity to Other VOCs (Ethanol and Acetone)

**Figure 7:**
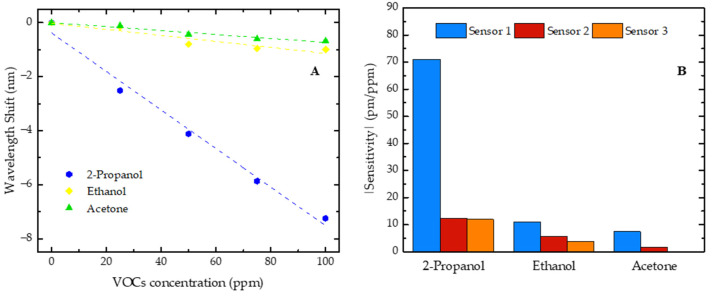
(**A**) Wavelength shift of sensor 1 for 2-propanol, ethanol, and acetone with concentrations increasing from 0 to 100 ppm; (**B**) sensitivity absolute value comparison of three sensors in response to 2-propanol, ethanol, and acetone.

The cross-sensitivity tests were conducted to evaluate the selectivity of the sensors to 2-propanol, with ethanol and acetone being selected due to their potential as disease biomarkers and their likely presence in biological samples. In addition to the characterization with 2-propanol, all the developed sensors were evaluated with two other VOCs, ethanol and acetone, at concentrations ranging from 0 to 100 ppm in steps of 25 ppm. The comparison of the sensitivities obtained by the sensors for the three VOCs under study is presented in Figure 7, with emphasis on the sensor with the highest sensitivity for these compounds (sensor 1).

The shifts in the resonant dip, occurring in opposite directions, were also observed with the increase in ethanol and acetone concentrations, where the sensor 1 resonant dip shifted towards shorter wavelengths (blue shift), while the resonant dips of sensors 2 and 3 shifted towards longer wavelengths (red shift). These results suggest that the observed phenomenon depends on the FP cavity size rather than on the VOC nature. Thus, all the sensitivities of sensor 1 are negative, while the sensitivities of sensors 2 and 3 are positive. However, for comparison purposes, in Figure 7B, we present the modulus of the sensitivity values.

Additionally, it was verified that each developed sensor was consistently more sensitive to 2-propanol, followed by ethanol, and least sensitive to acetone. It should also be added that, for sensor 3, no trend of wavelength shift evolution was observed when it was exposed to increasing concentrations of acetone. This higher sensitivity to 2-propanol might be related to the affinity of this polymer for this compound. The chemical structure of 2-propanol (a secondary alcohol) can allow specific interactions with the NOA88 polymer that do not occur as efficiently with ethanol (a primary alcohol) or acetone (a ketone).

### 4.4. Performance Evaluation of the Sensor with the Highest Sensitivity

Sensor 1, which demonstrated higher sensitivity to 2-propanol, was further validated. When compared to the literature, we can assert that we were working with a competitive interferometric sensor in terms of sensitivity to 2-propanol, as presented in Table 1.

**Figure 8:**
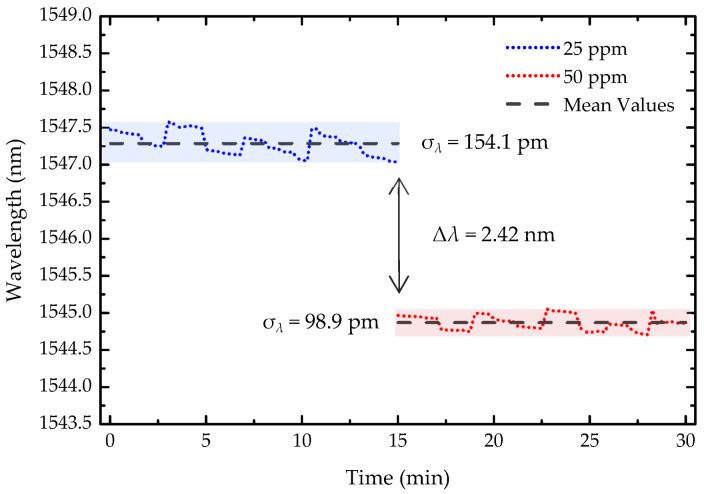
Stability of the sensor over 30 min for 25 ppm and 50 ppm of 2-propanol. The shaded areas represent the standard deviation (σ) for each concentration. Δ*λ* denotes the wavelength variation between the two concentration levels.

To evaluate the stability and resolution of the light-guided polymerization microtip-based OFS, the wavelength response was monitored over 30 min (Figure 8), with the spectrum recorded every 15 s, for two consecutive concentration steps of 25 ppm and 50 ppm. The resolution, δc, was calculated using the following equation:

(4) $$\delta c=\frac{{2\sigma }_{\lambda }\Delta c}{\Delta \lambda }$$

where σ*_λ_* represents the maximum wavelength standard deviation, which, in this case, was 154.1 pm. Δ*c* refers to the change in 2-propanol concentration between the two steps, 25 ppm, and Δ*λ* denotes the wavelength shift. With this, the sensor resolution was determined to be 3.18 ppm.

**Figure 9:**
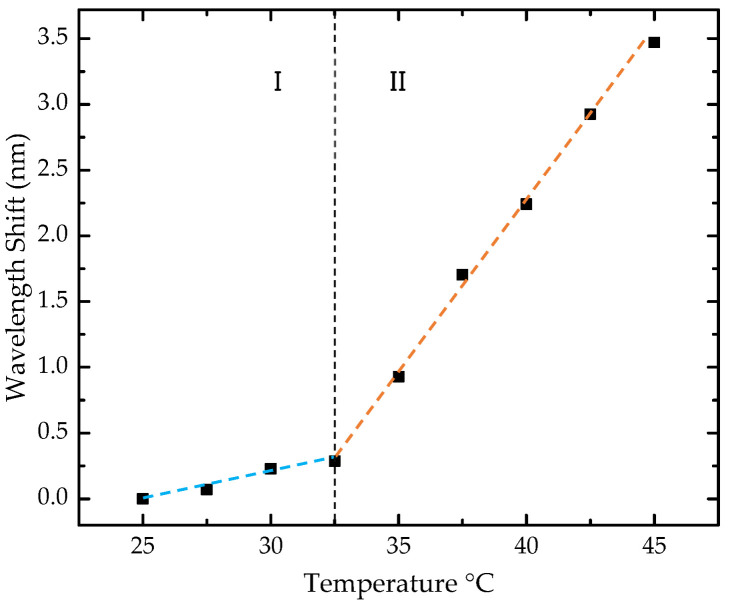
Wavelength shift of the sensor with temperature. The black squares represent the experimental data, while the two dashed lines, shown in distinct colors, indicate the linear fit applied to the two distinct sensitivity regions. Region I corresponds to the wavelength shift between 25 °C and 32.5 °C, and Region II corresponds to the wavelength shift between 32.5 °C and 45 °C.

The sensor cross-sensitivity to temperature was also assessed (Figure 9). The analysis involved nine temperature steps, ranging from 25 to 45 °C, with increments of 2.5 °C. A nonlinear behavior was observed in the studied range, with a consistent red shift toward longer wavelengths. To estimate the sensitivities, the range was divided into two regions, and a linear approximation was made to the experimental data for each segment.

In the range of 25 to 32.5 °C, a sensitivity of 40.1 ± 5.4 pm/°C was obtained, while in the range of 32.5 to 45 °C, the sensitivity was 256.5 ± 3.4 pm/°C. In our case, considering the temperature range near laboratory conditions, below 32.5 °C, corresponding to zone I of sensitivity, a low cross-sensitivity of 0.57 ppm of 2-propanol/°C was estimated by directly converting the value of 40.1 ± 5.4 pm/°C into 2-propanol concentration for each °C in temperature variation. Therefore, strict temperature control is not required during measurements.

## 5. Conclusions

A fiber-optic FP interferometric sensor was developed for the detection of 2-propanol using a light-guided induced microtip polymerization technique. The key features of this sensor include an easy and cost-effective fabrication process, high sensitivity for 2-propanol at room temperature, and good stability and resolution, with no significant hysteresis effects. The sensor performance is dependent on the length of the FP cavity, and the best results are obtained with a microtip length of approximately 15 µm. The sensor’s competitive performance makes it a promising tool for detecting 2-propanol in the gas phase. This sensor development addresses the growing demand for highly sensitive and precise VOC sensors. It holds significant potential for new biomedical applications, particularly in non-invasive disease diagnosis, as 2-propanol has shown promise as a biomarker in clinical samples. With the described fabrication method, the microtip length can be predicted with some degree of control. In the future, efforts could focus on further improving the fabrication process to achieve greater precision in obtaining the desired microtip length and ensuring higher reproducibility.

## Figures and Tables

**Table 1:** Performance comparison of the FP fiber-optic 2-propanol sensors.

Year	Sensing Layer	Sensing Structure	Sensitivity to 2-Propanol	Operating Range	Ref.
2016	PDMS	Cascaded fiber sensor	1.61 pm/ppm	0–7200 ppm	[34]
2016	Zeolite	Spherical end	910 pm/ppm	0–90 ppm	[35]
2017	Zeolite	Spherical end	4.99 pm/ppm	0–70 ppm	[14]
2019	PDMS	Cascaded fiber sensor	20 pm/ppm	0–500 ppm	[13]
	NOA88	Microtip	71.1 pm/ppm	0–100 ppm	Proposed sensor

## Data Availability

Data are contained within the article.
